# Vitamin K status and vascular calcification biomarkers as determinants of carotid plaque in peritoneal dialysis: a prospective study

**DOI:** 10.1080/0886022X.2026.2691345

**Published:** 2026-06-22

**Authors:** Irem Pamuk, Taha Enes Cetin, Beyza Hilal Kindan, Omer Faruk Akcay, Saliha Yildirim, Halit Nahit Sendur, Mahi Nur Cerit, Sevim Gonen, Burak Mert Akhan, Ulver Derici, Galip Guz, Ozant Helvaci

**Affiliations:** a Department of Internal Medicine, Faculty of Medicine, Gazi University, Ankara, Turkey; b Division of Nephrology, Department of Internal Medicine, Faculty of Medicine, Gazi University, Ankara, Turkey; c Department of Nephrology, Ministry of Health, Dr. Nafiz Korez Sincan State Hospital, Ankara, Turkey; d Department of Radiology, Faculty of Medicine, Gazi University, Ankara, Turkey; e Department of Pediatrics, Faculty of Medicine, Gazi University, Ankara, Turkey

**Keywords:** Peritoneal dialysis, vitamin K deficiency, PIVKA-II, vascular calcification, carotid plaque, matrix Gla protein

## Abstract

**Background:**

Vitamin K deficiency impairs the activation of calcification inhibitors such as matrix Gla protein, promoting vascular calcification in CKD. Protein induced by vitamin K absence-II (PIVKA-II) is a filtration-independent marker of vitamin K status validated in hemodialysis, yet no data exist in peritoneal dialysis (PD).

**Methods:**

Sixty prevalent PD patients at a single tertiary center were prospectively followed for 1 year. Serum PIVKA-II, dephosphorylated uncarboxylated matrix Gla protein (dp-ucMGP), and bone morphogenetic protein-2 (BMP-2) were measured at baseline and 12 months. Carotid plaque was assessed by ultrasonography. Independent predictors of plaque were identified by multivariable logistic regression and ROC analysis.

**Results:**

Carotid plaque was present in 36 patients (60%). Vitamin K deficiency (PIVKA-II >40 mAU/mL) was identified in 43.3% and was more prevalent in plaque-positive patients (55.5% vs 25.0%; *p* = 0.019). All three biomarkers were significantly elevated in the plaque group. Age (OR 1.26; 95% CI 1.07–1.42; *p* = 0.004) and PIVKA-II (OR 1.71; 95% CI 1.09–2.70; *p* = 0.022) independently predicted plaque. The area under the ROC curve was 0.766 (*p* = 0.001) with an optimal cutoff of 35.09 mAU/mL (sensitivity 86.1%, specificity 50.0%). Over 12 months, plaque prevalence rose to 70%; baseline BMP-2 was the sole predictor of new plaque development (*p* = 0.022).

**Conclusion:**

PIVKA-II–assessed vitamin K deficiency is common in PD and independently associated with carotid plaque. A threshold of 35 mAU/mL may be more sensitive than the conventional 40 mAU/mL cutoff, supporting PIVKA-II screening and targeted supplementation trials in PD.

## Introduction

Cardiovascular mortality accounts for roughly half of all deaths in patients receiving dialysis, a rate that far exceeds that of the age-matched general population [1]. Vascular calcification (VC) is central to this excess risk and, in chronic kidney disease (CKD), is driven not only by traditional factors but also by the depletion of endogenous calcification inhibitors [2].

Among these inhibitors, matrix Gla protein (MGP) stands out as the most potent local suppressor of arterial mineralization [3]. MGP requires vitamin K–dependent γ-carboxylation to function; in its absence, inactive dephosphorylated uncarboxylated MGP (dp-ucMGP) accumulates and can no longer antagonize bone morphogenetic protein-2 (BMP-2), an osteogenic signal that promotes vascular smooth muscle cell transdifferentiation and calcification [4,5].

Assessing vitamin K status in kidney disease, however, is not straightforward. Serum phylloquinone fluctuates with recent dietary intake and lipid levels, whereas dp-ucMGP reflects only the vascular compartment. Protein induced by vitamin K absence-II (PIVKA-II), the undercarboxylated form of prothrombin, provides a functional, glomerular filtration rate–independent measure of global vitamin K sufficiency and has been validated in end-stage kidney disease populations [6,7]. In a recent CKD cohort, Nyvad et al. demonstrated that PIVKA-II—but not dp-ucMGP—was independently associated with aortic calcification, further supporting the clinical relevance of this biomarker [8].

Subclinical vitamin K deficiency is remarkably common in hemodialysis (HD) cohorts, with reported prevalences of 82–97% when assessed by PIVKA-II [9,10]. Data in peritoneal dialysis (PD) patients are far more limited: studies using dp-ucMGP have documented elevated levels in the vast majority of PD patients and linked them to VC and adverse outcomes [11–13], but none has employed PIVKA-II. Whether the findings from HD populations apply to PD patients—who have distinct dietary patterns, longer preservation of residual renal function, and different uremic solute profiles—remains unknown.

We designed this prospective study to characterize vitamin K deficiency assessed by PIVKA-II in prevalent PD patients and to evaluate the association of PIVKA-II, dp-ucMGP, and BMP-2 with carotid artery plaque. Secondarily, we examined the 1-year longitudinal trajectory of these biomarkers and their relationship with new plaque development.

## Material and methods

### Study design and setting

This single-center prospective cohort study was conducted at the Department of Nephrology, Gazi University Faculty of Medicine, Ankara, Turkey, between September 2022 and September 2024. The study protocol was approved by the Gazi University Clinical Research Ethics Committee (approval no. 583; July 25, 2022) and conducted in accordance with the Declaration of Helsinki. All participants provided written informed consent.

### Participants

**Figure 1.**
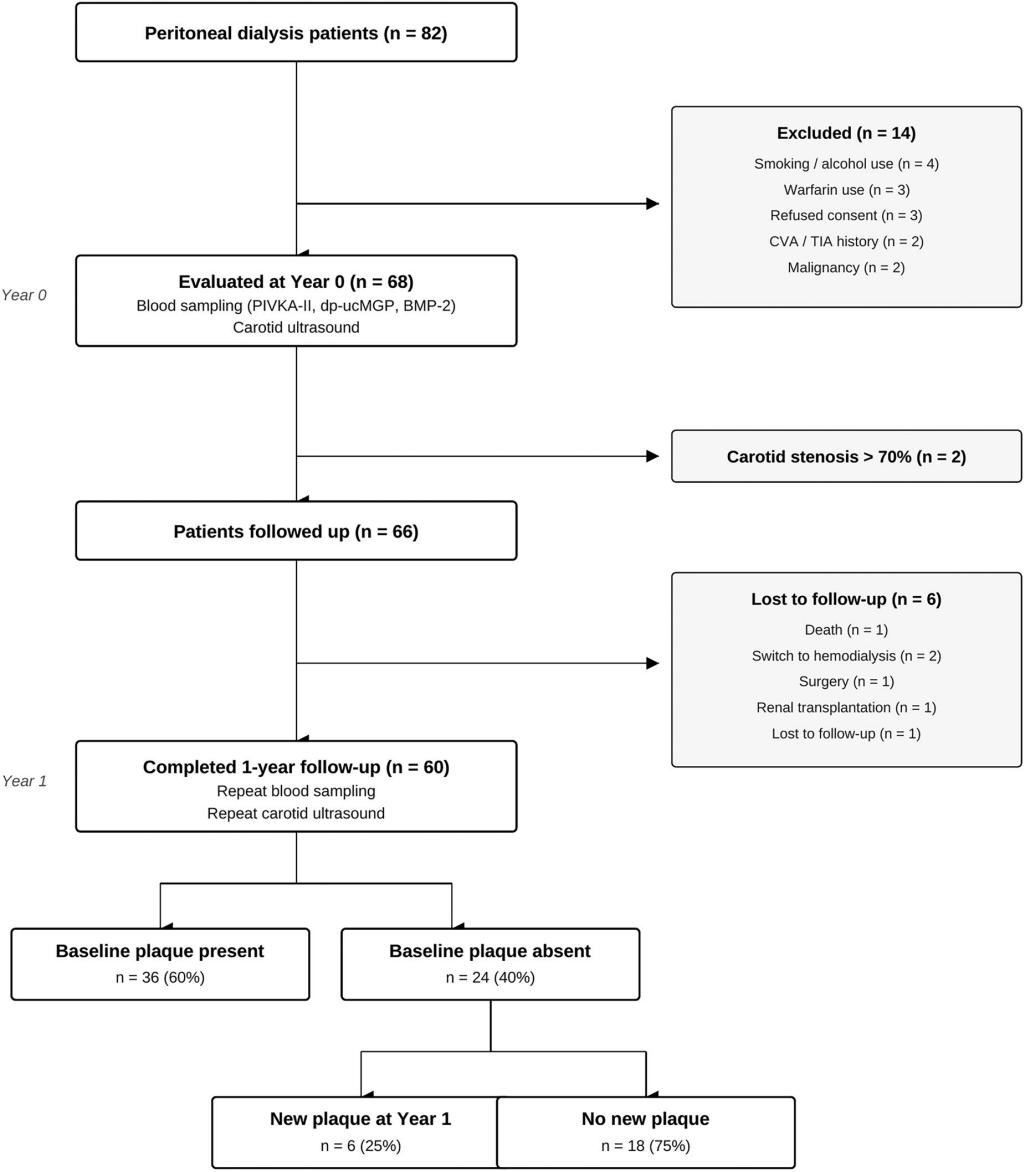
Flow diagram of patient selection and follow-up.

All adult patients receiving PD for at least 12 months at our center were assessed for eligibility. Exclusion criteria were: active malignancy or chronic systemic disease, current smoking or alcohol use, warfarin therapy, history of cerebrovascular accident or transient ischemic attack, carotid artery stenosis exceeding 70%, and refusal to participate. Of 82 patients assessed, 14 were excluded at screening and 2 additional patients were excluded after baseline carotid imaging revealed stenosis >70%, leaving 66 patients who entered follow-up. During the 1-year observation period, 6 patients were lost to follow-up (death, *n* = 1; switch to HD, *n* = 2; surgery, *n* = 1; renal transplantation, *n* = 1; loss to follow-up, *n* = 1), yielding a final cohort of 60 patients with complete baseline and 1-year data (Figure 1).

### Data collection

Demographic characteristics, primary cause of end-stage kidney disease, comorbidities, current medications, and PD-related parameters (modality, peritoneal equilibration test category, Kt/V, peritoneal creatinine clearance, residual renal function, and ultrafiltration volume) were recorded from medical charts at enrollment. Routine laboratory values including hemoglobin, serum creatinine, parathyroid hormone (PTH), calcium, phosphorus, lipid profile, and C-reactive protein were obtained from fasting morning blood samples drawn during scheduled clinic visits.

### Biomarker measurement

Fasting venous blood samples were collected at baseline and at 1 year into three separate 5 mL tubes. Samples were centrifuged at 3000 rpm for 5 min and serum aliquots were stored at −80 °C until analysis. PIVKA-II, dp-ucMGP, and BMP-2 were measured using commercially available enzyme-linked immunosorbent assay kits (Biont Biotechnology, Shanghai, China) according to the manufacturer’s instructions. Subclinical vitamin K deficiency was defined as a PIVKA-II level exceeding 40 mAU/mL, based on the upper limit of the 95% reference interval established in healthy populations [14].

### Carotid ultrasonography

Carotid ultrasonography was performed at baseline and at 1 year using a standardized protocol. Both common carotid arteries, carotid bifurcations, and internal carotid arteries were examined by the same experienced radiology team using an ultrasound device equipped with linear transducer. Carotid plaque was defined as a focal structure encroaching into the arterial lumen by at least 0.5 mm, or demonstrating a thickness greater than 50% of the surrounding intima-media thickness, or an intima-media thickness exceeding 1.5 mm, in accordance with the Mannheim carotid intima-media thickness and plaque consensus [15]. Plaque status was recorded as a binary outcome (present or absent). The same operator performed both the baseline and 1-year examinations to minimize inter-observer variability.

### Outcomes

The primary outcome was carotid plaque presence at baseline. Secondary outcomes included new plaque development during 1-year follow-up among patients without plaque at baseline and longitudinal changes in PIVKA-II, dp-ucMGP, and BMP-2 levels.

### Statistical analysis

Analyses were performed using SPSS version 26.0 (IBM Corp., Armonk, NY, USA). Continuous variables were expressed as mean ± standard deviation or median (interquartile range) as appropriate; categorical variables were reported as number (percentage). Distribution normality was assessed by the Kolmogorov–Smirnov test. Group comparisons were performed using the independent-samples t-test or Mann–Whitney U test for continuous variables, and the Pearson chi-square test for categorical variables. Paired comparisons between baseline and 1-year values were assessed using the paired-samples t-test or Wilcoxon signed-rank test.

Candidate variables were screened through univariate binary logistic regression for the primary outcome. Variables reaching statistical significance (*p* < 0.05) in univariate analysis were then entered simultaneously into a multivariable binary logistic regression model using the Enter method with Wald statistic, given the limited sample size.

Receiver operating characteristic curve analysis was performed for baseline PIVKA-II to evaluate its discriminative ability for carotid plaque. The AUC and 95% confidence interval were calculated. The optimal cutoff value was determined using Youden’s index (maximum sensitivity + specificity − 1).

A formal sample size calculation was not performed *a priori*, as no prior study has simultaneously evaluated these three biomarkers in PD patients, precluding reliable effect size estimation. The sample of 60 patients is within the range of comparable single-center PD biomarker studies.

A two-tailed *p*-value of less than 0.05 was considered statistically significant for all analyses.

## Results

### Baseline characteristics

Sixty PD patients (31 male; mean age 55.8 ± 14.5 years) with a mean dialysis vintage of 4.9 ± 2.9 years were analyzed. The majority were on automated PD (60%), and residual renal function was preserved in 85%. The most common causes of end-stage kidney disease were hypertension (36.7%) and diabetes mellitus (20%). Comorbid hypertension was present in 86.6%, coronary artery disease in 33.3%, and congestive heart failure in 28.3%. The mean body mass index was 27.2 ± 3.8 kg/m^2^. Statins were used by 73.3% and calcium-based phosphate binders by 50.0% of patients. Baseline demographics, laboratory values, medications, and PD-related characteristics are summarized in Table 1; PD adequacy parameters are provided in Supplementary Table S1.

**Table 1.** Baseline characteristics of the study population (*n* = 60).

Variable	Value
Demographics	
Age, years	55.8 ± 14.5
Male sex, *n* (%)	31 (51.7)
BMI, kg/m²	27.2 ± 3.8
PD duration, years	4.9 ± 2.9
Primary cause of ESKD, *n* (%)	
Hypertension	22 (36.7)
Diabetes mellitus	12 (20.0)
Glomerulonephritis	10 (16.7)
Unknown	8 (13.3)
Comorbidities, *n* (%)	
Hypertension	52 (86.6)
Coronary artery disease	20 (33.3)
Diabetes mellitus	18 (30.0)
Congestive heart failure	17 (28.3)
Peripheral artery disease	8 (13.3)
Medications, *n* (%)	
Calcium carbonate	26 (43.3)
Calcium acetate	4 (6.7)
Sevelamer	16 (26.7)
Calcitriol	16 (26.7)
Cinacalcet	3 (5.0)
Acetylsalicylic acid	24 (40.0)
P2Y12 inhibitors	4 (6.7)
PD modality, *n* (%)	
CAPD	24 (40.0)
APD	36 (60.0)
Residual renal function present, *n* (%)	51 (85.0)
Laboratory values	
Systolic blood pressure, mmHg	125.1 ± 19.9
Diastolic blood pressure, mmHg	71.0 ± 10.9
Hemoglobin, g/dL	11.2 ± 1.43
Creatinine, mg/dL	8.35 ± 3.02
PTH, pg/mL	583 ± 392
Calcium, mg/dL	9.07 ± 0.7
Phosphorus, mg/dL	5.1 ± 1.6
LDL cholesterol, mg/dL	106 ± 49
Triglycerides, mg/dL	167 ± 85.7
HDL cholesterol, mg/dL	41.08 ± 11.7
CRP, mg/L	7.85 ± 9.5

Data presented as mean ± SD or *n* (%).

Abbreviations: BMI, body mass index; ESKD, end-stage kidney disease; ADPKD, autosomal dominant polycystic kidney disease; PD, peritoneal dialysis; CAPD, continuous ambulatory PD; APD, automated PD; PTH, parathyroid hormone; LDL, low-density lipoprotein; HDL, high-density lipoprotein; CRP, C-reactive protein.

### Biomarker levels and vitamin K status by plaque presence

Carotid plaque was identified in 36 patients (60%) at baseline. Mean PIVKA-II was significantly higher in the plaque-positive group than in the plaque-negative group (44.9 ± 11.0 vs. 34.8 ± 6.4 mAU/mL; *p* = 0.001). Similarly, dp-ucMGP (840 ± 444 vs. 644 ± 372 pmol/L; *p* = 0.008) and BMP-2 (11.4 ± 6.4 vs. 9.45 ± 6.2 ng/mL; *p* = 0.046) were elevated in the plaque group, although the difference in BMP-2 was of borderline significance (Table 2). Vitamin K deficiency (PIVKA-II >40 mAU/mL) was present in 26 patients overall (43.3%) and was significantly more prevalent in the plaque-positive group (55.5% vs. 25.0%; *p* = 0.019).

**Table 2.** Biomarker levels and vitamin K deficiency status according to baseline carotid plaque Presence.

	Plaque (+) (*n* = 36)	Plaque (−) (*n* = 24)	*p*
PIVKA-II, mAU/mL	44.9 ± 11.0	34.8 ± 6.4	**0.001**
dp-ucMGP, pmol/L	840 ± 444	644 ± 372	**0.008**
BMP-2, ng/mL	11.4 ± 6.4	9.45 ± 6.2	**0.046**
Vitamin K deficiency, *n* (%)	20 (55.5)	6 (25.0)	**0.019**

Pearson chi-square test. Vitamin K deficiency defined as PIVKA-II >40 mAU/mL. Bold values indicate statistical significance (*p* < 0.05).

### Predictors of carotid plaque

On univariate binary logistic regression screening, age, baseline PIVKA-II, body mass index, and residual renal function were significantly associated with plaque presence (all *p* < 0.05). dp-ucMGP, BMP-2, sex, PD duration, PD modality, Kt/V, PTH, phosphorus, and coronary artery disease history did not reach significance and were not entered into the multivariable model. Baseline mineral metabolism parameters stratified by plaque status are presented in Supplementary Table S2. Although PTH and phosphorus levels were higher in the plaque-positive group in unadjusted comparisons, neither variable demonstrated an independent association with plaque in regression analyses. Categorical vitamin K deficiency status was excluded owing to collinearity with continuous PIVKA-II. When the four significant univariate predictors were entered simultaneously, only age (OR 1.255 per year; 95% CI 1.072–1.420; *p* = 0.004) and baseline PIVKA-II (OR 1.713 per mAU/mL; 95% CI 1.088–2.698; *p* = 0.022) retained independent significance, while body mass index (OR 0.766; 95% CI 0.528–1.111; *p* = 0.157) and residual renal function (OR 7.910; 95% CI 0.499–12.538; *p* = 0.100) did not (Table 3).

**Table 3.** Multivariable logistic regression analysis—predictors of baseline carotid plaque Presence.

Variable	OR	95% CI	*p*
Age (per year)	1.255	1.072–1.420	**0.004**
Baseline PIVKA-II (per mAU/mL)	1.713	1.088–2.698	**0.022**
BMI (per kg/m²)	0.766	0.528–1.111	0.157
Residual renal function (present vs absent)	7.910	0.499–12.538	0.100

Variables reaching significance on univariate screening were entered simultaneously (Enter method, Wald statistic). Bold values indicate statistical significance (*p* < 0.05).

### Discriminative performance of PIVKA-II

**Figure 2.**
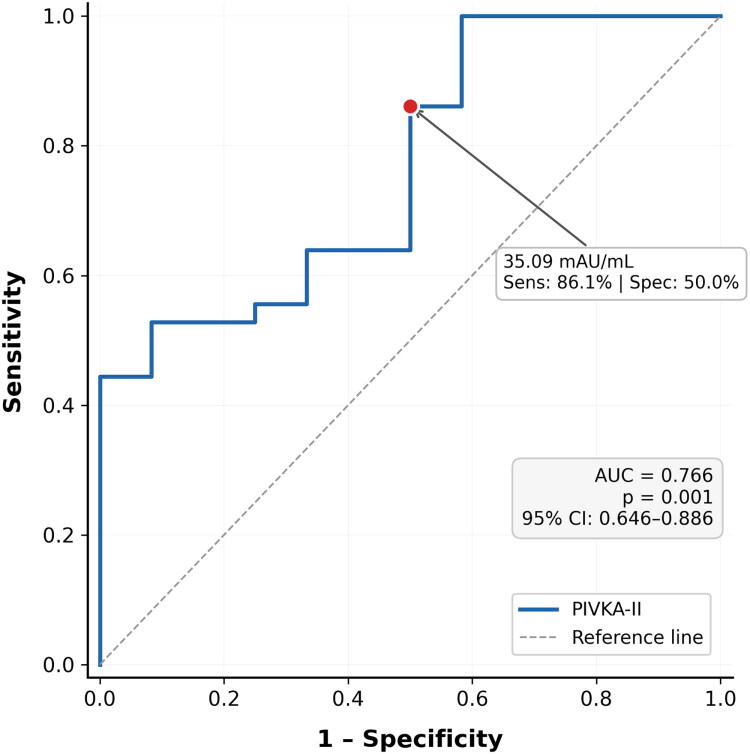
ROC curve of baseline PIVKA-II for carotid plaque.

ROC analysis yielded an AUC of 0.766 (95% CI 0.646–0.886; *p* = 0.001) for baseline PIVKA-II in identifying patients with carotid plaque (Figure 2). The optimal cutoff derived by Youden’s index was 35.09 mAU/mL (sensitivity 86.1%, specificity 50.0%), lower than the conventional 40 mAU/mL threshold.

### Longitudinal changes

Over 12 months, PIVKA-II increased significantly (40.86 ± 10.67 to 42.88 ± 11.42 mAU/mL; *p* = 0.001) and BMP-2 rose from 10.66 ± 6.36 to 12.39 ± 7.19 ng/mL (*p* = 0.037), while dp-ucMGP remained stable (762.31 ± 424 to 765.10 ± 359.75 pmol/L; *p* = 0.128; Supplementary Table S3). Plaque prevalence increased from 60% to 70%; among the 24 initially plaque-free patients, 6 (25%) developed new plaque at 1 year. Baseline BMP-2 was the only biomarker significantly higher in patients who developed new plaque compared to those who did not (15.6 ± 0.2 vs. 7.3 ± 5.8 ng/mL; *p* = 0.022). Neither baseline PIVKA-II (35.04 ± 5.80 vs. 34.74 ± 6.84 mAU/mL; *p* = 0.923) nor vitamin K deficiency status (*p* = 0.586) predicted new plaque development (Supplementary Table S4). Notably, the significant difference in BMP-2 between groups was no longer present at 1 year (*p* = 0.721).

## Discussion

In this prospective study of 60 PD patients, subclinical vitamin K deficiency assessed by PIVKA-II was present in 43% and was independently associated with carotid plaque after adjustment for age, body mass index, and residual renal function. All three biomarkers—PIVKA-II, dp-ucMGP, and BMP-2—were elevated in patients with plaque, yet only age and PIVKA-II retained significance in multivariable analysis. These findings suggest that the vascular impact of vitamin K deficiency in PD extends beyond any single downstream pathway.

The 43% deficiency rate in our cohort is notably lower than the 82–97% reported in HD populations using the same PIVKA-II threshold [9,10]. This difference is not unexpected. PD patients typically face fewer dietary potassium and phosphorus restrictions than their HD counterparts, potentially allowing greater intake of vitamin K–rich foods [6]. Furthermore, 85% of our cohort retained residual renal function, which may contribute to clearance of uremic solutes that interfere with vitamin K recycling [16]. Despite the lower prevalence relative to HD, a rate of 43% remains clinically important, exceeds that reported in healthy populations [14], and is broadly consistent with the limited PD data available using dp-ucMGP-based assessments [11,12].

The observation that PIVKA-II—but not dp-ucMGP or BMP-2—independently predicted plaque deserves particular attention. dp-ucMGP reflects the carboxylation status of a single vascular protein [17], and BMP-2 is a downstream osteogenic effector [5]. PIVKA-II, by contrast, indexes hepatic vitamin K reserves that supply all vitamin K–dependent proteins, including not only MGP but also Gas6, osteocalcin, protein S, and other Gla-domain proteins with anti-calcific or anti-inflammatory functions [6,18,19]. Beyond its established role in bone metabolism, osteocalcin is increasingly recognized as a hormonally active protein involved in systemic metabolic, inflammatory, and vascular regulation, supporting the concept that vitamin K deficiency may exert multisystem biological effects extending beyond VC pathways alone [20,21]. PIVKA-II may therefore capture the cumulative biological cost of vitamin K deficiency more completely than any single-pathway marker. A similar hierarchy was recently observed by Nyvad et al. who found that PIVKA-II but not dp-ucMGP was associated with aortic calcification in a CKD cohort [8]. Together, these data point to a broader role for vitamin K deficiency in vascular disease than the MGP-centric model alone would predict.

ROC analysis identified an optimal PIVKA-II cutoff of 35.09 mAU/mL (sensitivity 86.1%, specificity 50.0%), below the conventional 40 mAU/mL threshold used in most studies [14]. We regard this finding as exploratory. The lower cutoff may reflect assay-specific characteristics of the ELISA platform used, differences in population vitamin K intake, or the possibility that vascular harm begins at a lower degree of deficiency than currently recognized. Regardless of the precise threshold, the clinical message is consistent: a substantial proportion of PD patients have vitamin K insufficiency that current cutoffs may underestimate. External validation in independent PD cohorts is essential before any clinical application. The 35 mAU/mL cutoff is data-derived and hypothesis-generating; it should not be adopted clinically without independent confirmation. Nevertheless, the identified cutoff demonstrated acceptable discriminative performance in our cohort and may serve as a useful preliminary threshold for future validation studies and prospective investigations in PD populations.

Over 12 months, PIVKA-II and BMP-2 increased significantly while dp-ucMGP remained stable, and plaque prevalence rose from 60% to 70%. Among the 24 initially plaque-free patients, six (25%) developed new plaque—to our knowledge the first report of 1-year carotid plaque incidence in PD. The only baseline biomarker that differed between patients who did and did not develop new plaque was BMP-2. As BMP-2 is the most proximate effector of osteogenic vascular smooth muscle cell transdifferentiation [22,23], it may reflect an active calcification process already underway, whereas PIVKA-II and dp-ucMGP signal a permissive milieu rather than an immediate event. The small subgroup size (six events among 24 patients) precludes definitive conclusions and warrants cautious interpretation of the longitudinal findings; therefore, longer follow-up with larger cohorts will be needed to clarify the predictive role of each biomarker for incident plaque.

These findings have direct relevance for vitamin K supplementation trials in dialysis. A recent meta-analysis showed that vitamin K2 reduced circulating dp-ucMGP but failed to attenuate radiological calcification scores [24]. The Trevasc-HDK trial similarly demonstrated effective dp-ucMGP reduction without change in coronary or valvular calcification [25]. A common limitation of these trials is the assumption that all dialysis patients are vitamin K–deficient, without baseline biochemical confirmation [6]. If deficiency is present in roughly half—rather than virtually all—PD patients, unselected supplementation will dilute any treatment effect. Importantly, recent data confirm that MK-7 supplementation effectively reduces PIVKA-II in dialysis patients without procoagulant risk [26], supporting the feasibility of a targeted approach. PIVKA-II-based screening could identify the subgroup most likely to benefit, offering a path toward more efficient trial design. The ongoing VIKIPEDIA trial, a PD-specific randomized study incorporating baseline vitamin K assessment, represents an important step in this direction [27].

This study has several strengths. It is the first to apply PIVKA-II to a PD population and the first to measure three complementary biomarkers of the vitamin K–calcification axis against a structural vascular endpoint in this modality. Strict exclusion of smokers, warfarin users, and patients with prior cerebrovascular events reduced confounding. The prospective design with paired biomarker and imaging assessments enabled both cross-sectional and longitudinal analysis, including the first estimate of new plaque incidence in PD.

Limitations should be acknowledged. The single-center design and sample size of 60 constrain generalizability and statistical power, particularly for the incident-plaque subanalysis. One year of follow-up may be insufficient to capture the slow progression of VC [2]. Dietary vitamin K intake was not assessed, so we cannot determine the contribution of intake versus metabolic consumption to the observed deficiency. In addition, detailed prospective dietary data related to phosphate restriction and CKD-related nutritional management were not systematically collected during follow-up. PIVKA-II was measured by ELISA rather than chemiluminescent immunoassay, which limits direct comparison with hepatology ­literature [28]. The proposed 35 mAU/mL cutoff requires external validation before clinical implementation.

## Conclusion

Subclinical vitamin K deficiency assessed by PIVKA-II affects a substantial proportion of PD patients and is independently associated with carotid artery plaque. The independent predictive value of PIVKA-II over pathway-specific markers such as dp-ucMGP suggests that the vascular consequences of vitamin K deficiency are not limited to MGP inactivation alone. These pilot data support the use of PIVKA-II as a screening tool to identify vitamin K–deficient PD patients who may benefit most from targeted supplementation, and they provide the rationale for larger, multicenter validation studies with hard cardiovascular endpoints.

## Supplementary Material

Supplementary Tables.docx

## Data Availability

The datasets generated and/or analyzed during the current study are available from the corresponding author on reasonable request.
