# Supplementary material for: Vitamin K status and vascular calcification biomarkers as determinants of carotid plaque in peritoneal dialysis: a prospective study
Source: Ren Fail. 2026 Jun 22;48(1):2691345. doi: 10.1080/0886022X.2026.2691345 (PMC13288904; doi:10.1080/0886022X.2026.2691345)
Supplement: Supplementary Tables.docx [file IRNF_A_2691345_SM5932.docx]

**SUPPLEMENTARY MATERIAL**

*Vitamin K Status and Vascular Calcification Biomarkers as Determinants of Carotid Plaque in Peritoneal Dialysis: A Prospective Study*

Irem Pamuk, Taha Enes Cetin, Ozant Helvaci

**Supplementary Table S1. Peritoneal Dialysis Adequacy Parameters (n = 60)**

| **Parameter** | **Value** |
| --- | --- |
| **PET category, n (%)** |  |
| Low | 4 (6.7) |
| Low-average | 36 (60.0) |
| High-average | 6 (10.0) |
| High | 14 (23.3) |
| **Adequacy parameters** |  |
| Peritoneal CrCl, L/week/1.73 m² | 72.26 ± 31.1 |
| Kt/V | 2.16 ± 0.67 |
| Residual renal function, mL/day | 1098 ± 638 |
| Ultrafiltration volume, mL/day | 777.5 ± 533 |
| **Dialysis solutions used, n (%)** |  |
| 1.5% dextrose | 38 (63.3) |
| 2.5% dextrose | 38 (63.3) |
| 4.5% dextrose | 2 (3.3) |
| Icodextrin | 28 (46.7) |

Data presented as mean ± SD or n (%).

*Abbreviations: PET, peritoneal equilibration test; CrCl, creatinine clearance; Kt/V, dialysis adequacy index.*

**Supplementary Table S2. Mineral Metabolism Parameters by Baseline Carotid Plaque Status (n = 60)**

| **Parameter** | **Plaque (+) (n = 36)** | **Plaque (−) (n = 24)** | **p** |
| --- | --- | --- | --- |
| PTH, pg/mL | 795.2 ± 395.4 | 442.0 ± 324.4 | **<0.005** |
| Calcium, mg/dL | 9.09 ± 0.93 | 9.06 ± 0.59 | 0.89 |
| Phosphorus, mg/dL | 5.68 ± 1.9 | 4.71 ± 1.2 | **0.021** |

Data presented as mean ± SD. Compared by independent-samples t-test.

*Abbreviations: PTH, parathyroid hormone.*

**Supplementary Table S3. Longitudinal Changes in Biomarkers and Mineral Metabolism Parameters — Year 0 vs Year 1 (n = 60)**

| **Biomarker** | **Year 0** | **Year 1** | **p** |
| --- | --- | --- | --- |
| PIVKA-II, mAU/mL | 40.86 ± 10.67 | 42.88 ± 11.42 | **0.001** |
| dp-ucMGP, pmol/L | 762.31 ± 424 | 765.10 ± 359.75 | 0.128 |
| BMP-2, ng/mL | 10.66 ± 6.36 | 12.39 ± 7.19 | **0.037** |
| Calcium, mg/dL | 9.07 ± 0.73 | 9.01 ± 0.72 | 0.942 |
| Phosphorus, mg/dL | 5.10 ± 1.60 | 5.02 ± 1.08 | 0.858 |
| PTH, pg/mL | 583 ± 392 | 542 ± 399 | 0.469 |

Carotid plaque prevalence: Year 0, 36/60 (60%) → Year 1, 42/60 (70%). New plaque development in initially plaque-free patients: 6/24 (25%).

Data presented as mean ± SD. Paired comparison by Wilcoxon signed-rank test.

*Abbreviations: PIVKA-II, protein induced by vitamin K absence-II; dp-ucMGP, dephosphorylated uncarboxylated matrix Gla protein; BMP-2, bone morphogenetic protein-2, PTH, parathyroid hormone.*

**Supplementary Table S4. Baseline Biomarker Levels in Patients With vs Without New Plaque Development (Initially Plaque-Free Subgroup, n = 24)**

| **Biomarker** | **New plaque (+) (n = 6)** | **New plaque (−) (n = 18)** | **p** |
| --- | --- | --- | --- |
| PIVKA-II, mAU/mL | 35.04 ± 5.80 | 34.74 ± 6.84 | 0.923 |
| dp-ucMGP, pmol/L | 518.2 ± 118.4 | 687.5 ± 418.3 | 0.494 |
| BMP-2, ng/mL | 15.6 ± 0.2 | 7.3 ± 5.8 | **0.022** |

**Vitamin K deficiency status and new plaque development (Chi-square):**

|  | **New plaque (+) n (%)** | **New plaque (−) n (%)** | **p** |
| --- | --- | --- | --- |
| Vitamin K deficient (PIVKA-II >40 mAU/mL) | 2 (33.3) | 4 (22.2) | 0.586 |
| Vitamin K sufficient (PIVKA-II ≤40 mAU/mL) | 4 (66.7) | 14 (77.8) |  |

Data presented as mean ± SD or n (%). Continuous variables compared by Mann–Whitney U test.

*Abbreviations: as in Supplementary Table S2.*
